# Unmet needs and related factors of Korean breast cancer survivors: a multicenter, cross-sectional study

**DOI:** 10.1186/s12885-019-6064-8

**Published:** 2019-08-27

**Authors:** Byung Joo Chae, Jihyoun Lee, Se Kyung Lee, Hyuk-Jae Shin, So-Youn Jung, Jong Won Lee, Zisun Kim, Min Hyuk Lee, Juhyung Lee, Hyun Jo Youn

**Affiliations:** 1 Department of Surgery, Samsung Medical Center, Sungkyunkwan University school of Medicine, Seoul, Korea; 2 Department of Surgery, Soonchunhyang University Seoul Hospital, Soonchunhyang University College of Medicine, Seoul, Korea; 3 Department of Surgery, Myongji Hospital, Goyang, Korea; 4 Breast Cancer Center, National Cancer Center, Goyang, Korea; 5 Department of Surgery, Asan Medical Center, University of Ulsan College of Medicine, Seoul, Korea; 6 Department of Surgery, Soonchunhyang University Bucheon Hospital, Soonchunhyang University College of Medicine, Bucheon, Korea; 7 Department of Preventive Medicine, Chonbuk National University Medical School, Jeonju, Korea; 8 Department of Surgery, Research Institute of Clinical Medicine, Chonbuk National University Hospital, Chonbuk National University and Biomedical Research Institute, 20, Geonji-ro, Deokjin-gu, Jeonju-si, Jeollabuk-do Korea

**Keywords:** Unmet need, Breast neoplasm, Survivor

## Abstract

**Background:**

Identification of specific needs in patients with cancer is very important for the provision of patient-centered medical service. The aim of this study was to investigate the unmet needs and related factors of Korean breast cancer survivors.

**Methods:**

A multicenter, cross-sectional, interview survey was performed among 332 Korean breast cancer survivors. The Comprehensive Needs Assessment Tool for cancer patients was administered to survivors who gave written informed consent to participate. Data were analyzed using t-test, ANOVA and multiple regression analysis.

**Results:**

The level of unmet needs was highest in the domain ‘Information and education’ (mean ± SD; 1.70 ± 1.14) and the item with the highest level of unmet needs was ‘Needed help in coping with fear of recurrence’ (2.04 ± 1.09). Unmet needs were correlated with age, stage, multiplicity, HER2, treatment state, marital status, employment, psychosocial status, and problems in EQ-5D dimensions. In multiple regression analysis, the 50–59 age group showed a higher level of recognition for physical symptom needs and the unemployed group expressed greater needs for information and education. Survivors with multiplicity had greater needs in the domains of healthcare staff and physical symptom. The stress group showed high levels of needs in all domains except religious support. The group with thoughts of suicide showed higher levels of unmet needs for physical symptom.

**Conclusion:**

Most prevalent unmet needs in Korean breast cancer survivors were found in the ‘information and education’ domain. The 50–59 age group, unemployment, multiplicity, stress and suicidal thoughts were associated with higher levels of unmet needs among Korean breast cancer survivors. Our findings revealed more vulnerable breast cancer survivors with unmet needs and physicians should take a precision approach to satisfy unmet needs of these survivors.

**Electronic supplementary material:**

The online version of this article (10.1186/s12885-019-6064-8) contains supplementary material, which is available to authorized users.

## Background

According to the GLOBOCAN report, 18.1 million new cancer cases and 2.1 million (11.6%) breast cancer cases were projected to occur in 2018 [1]. Among females, breast cancer is the most commonly diagnosed cancer in the vast majority of the countries, accounting for almost 1 in 4 cancer cases. The number of cancer survivors is increasing because the earlier detection and advances in treatment have improved the survival rates of cancer patients. Cancer survivors in Korea are 1.7 million people and breast cancer survivors among them are about 198,000 (11.4%), representing the fourth largest group among the total cancer survivors [2]. In the situation where over 86% of Korean breast cancer survivors live more than 10 years after the diagnosis, identifying and satisfying their unmet needs have become an important issue.

The current trends in modern medicine include a shift from a disease-centered model where physicians make all the decisions about the overall aspects of medical services to a patient-centered model where patients actively participate in decisions about diagnosis and treatment and patients’ preferences and needs are considered and reflected in medical services. However, there is still a lack of interest in all aspects of the patient as a whole and holistic care is still being neglected [3]. Accurate identification and improvement of unmet needs of cancer survivors not only increase the satisfaction of survivors, but they can also improve the quality of cancer-related medical services. In addition, identifying the factors that affect the unmet needs of cancer survivors is a very important element in the provision of appropriate medical services for them and efficient distribution of medical resources [4].

Major unmet needs of cancer survivors include informational, psychological, physical and social support needs. Informational needs can be attributed to the anxiety and fear of most cancer patients due to a lack of adequate information about their disease and treatment and psychological needs are known to stem from the inability to efficiently cope with the psychological burden generated from the time of cancer diagnosis [5]. Another type of important unmet needs is physical needs and they involve many side effects of cancer treatment, such as nausea, vomiting, sleep disorder, alopecia, and myalgia. These physical difficulties cause many problems of psychological aspects and lead to a decline in quality of life (QoL) among cancer survivors. Active provision of social support for cancer survivors has been shown to not only induce psychosocial adaptation and health promotion behavior but also have a positive effect on the increase of the survival rate [6].

To provide appropriate medical services for cancer survivors, efforts should be made to identify the factors that affect unmet needs as well as specific elements of unmet needs. So far, patients’ characteristics, cancer type, stage, treatment modality and time of diagnosis have been presented as the factors affecting the unmet needs of cancer survivors [7–9].

As described above, identifying the unmet needs of cancer survivors and analyzing the related factors are very important for the overall improvement of cancer management. However, to date, most studies on unmet needs have been limited to the analysis of a single factor of unmet needs from a partial point of view and very few studies have dealt with the overall aspects of specific cancer survivors comprehensively.

Therefore, this study aimed to clarify major elements of unmet needs and factors influencing unmet needs from a comprehensive perspective among Korean breast cancer survivors and analyze the relationships between the attributes in order to provide a basis for provision of appropriate medical services for breast cancer survivors.

## Methods

### Study population

From July 2016 to August 2017, we conducted a cross-sectional interview survey with 332 participants who voluntarily agreed to participate in the study and signed the informed consent form among the patients who were diagnosed with breast cancer at six medical centers (Chonbuk National University Hospital, National Cancer Center, Samsung Medical Center, Myongji Hospital, Soonchunhyang University Seoul and Bucheon Hospital) in Korea. Electronic medical records were used to analyze the clinical and pathological factors of subjects and research was conducted after obtaining the approval from the institutional review board of each center. Of the total participants, 320 people (96.1%) were included in the final analysis excluding 12 respondents with missing data. Among sociodemographic characteristics of study subjects, income was the average monthly income of household and we investigated into before and after cancer diagnosis. Attendance of self-help group only included actual meeting except online participation. Stress was assessed using the question ‘How much stress do you perceive in your daily life?’ and answers ‘little’, ‘a little’, ‘high’ and ‘very high’. Despair and thought of suicide were evaluated with the following question: ‘Have you ever felt serious despair sequentially for more than two weeks in the past year?’ and ‘Have you ever thought about dying in the past year?’

### Measurements

The needs of breast cancer survivors were assessed by the Comprehensive Needs Assessment Tool (CNAT), which was developed at Korea’s National Cancer Center [10]. CNAT is the most appropriate tool to measure the needs of Korean cancer survivors which takes into consideration sociocultural aspects. CNAT consists of 59 items, clustered into 8 domains: information and education (10 items), psychological problem (10 items), health care staff (8 items), physical symptom (12 items), hospital service (6 items), family/personal relation problem (3 items), religious/spiritual support (2 items), and social support (8 items) (Additional file 1: Table S1). Subjects responded to each item on a 4-point Likert scale (0 = no need, 1 = low need, 2 = moderate need, 3 = high need) based on their experience of the past one month.

QoL was assessed using the EQ-5D validated among Korean subjects, and it was measured using a three-point scoring system (1 = no problems; 2 = some problems; 3 = severe problems) in five domains: subject’s mobility, self-care, usual activities, pain/discomfort and anxiety/depression [11]. Subjects were classified into two groups according to the responses to the questions of the EQ-5D: survivors who had one or more problems and those who had no problems in those areas.

### Statistical analyses

Principle component analysis was used for factor extraction. The eigenvalue was defined as 1 and factor analysis was performed using an orthogonal rotation by the varimax method. T-test and ANOVA were used to compare the need scores for each factor according to the demographic and other characteristics of breast cancer survivors. Multiple regression analysis was conducted to examine the influences of major variables on the level of needs. In multiple regression analysis, the need scores for each factor were used as dependent variables and variables which were shown to be statistically significant by univariate analysis were used as independent variables. All statistical analyses were performed using SPSS version 23.0 (SPSS Inc., Chicago, IL, USA) and the level of statistical significance was set at *p* < 0.05.

## Results

### Characteristics of the study population

The age at the time of surgery for breast cancer was 40~59 years in 252 people (75.9%), the time since diagnosis was 1~3 years in 48.2% (160/332 people). 67.8% (225/332 people) received breast conserving surgery and the majority of the subjects (290 people, 87.4%) were early breast cancer survivors of stage II or below. The positive rates of estrogen receptor, progesterone receptor and HER2 were 78.3, 72.3 and 28.6%, respectively. There were 79 cases (23.8%) with co-morbidities such as hypertension and diabetes, 141 cases (42.5%) of patients receiving adjuvant treatment such as chemotherapy, radiation and target therapy after surgery, 144 cases (43.4%) of patients receiving regular radiological examination after adjuvant treatment, and 47 other cases (14.1%) (palliative treatment for recurrence, complementary treatment or simple follow-up) (Table 1).

**Table 1:** Clinicopathological characteristics of study subjects

Characteristics	Number (%)
Age (years)	
< 40	44 (13.3)
40 ~ 49	129 (38.9)
50 ~ 59	123 (37.0)
≥ 60	36 (10.8)
Menopausal status	
Postmenopause	127 (38.3)
Time since diagnosis (years)	
≤ 1	73 (22.0)
> 1, ≤ 3	160 (48.2)
> 3, ≤ 5	66 (19.9)
> 5	33 (9.9)
Family history of breast cancer	
Yes	27 (8.1)
Operation method of breast	
BCS	225 (67.8)
Mastectomy	79 (23.8)
(NA)SSM	28 (8.4)
Operation method of axilla	
SLNB	212 (63.9)
ALND	120 (36.1)
TNM stage	
0	13 (3.9)
I	129 (38.9)
II	148 (44.6)
III & IV	42 (12.6)
Multiplicity	
Yes	62 (18.7)
Estrogen receptor	
Positive	260 (78.3)
Progesterone receptor	
Positive	240 (72.3)
HER2	
Positive	95 (28.6)
Chemotherapy	
Yes	248 (74.7)
Hormonal therapy	
Yes	236 (71.1)
Radiation therapy	
Yes	262 (78.9)
Target therapy	
Yes	72 (21.7)
Co-morbidity	
Yes	79 (23.8)
Adjuvant treatment	
During	141 (42.5)
After	144 (43.4)
Others^a^	47 (14.1)

Abbreviations: *BCS* breast conserving surgery, (*NA*) (nipple areolar), *SSM* skin sparing mastectomy, *SLNB* sentinel lymph node biopsy, *ALND* axillary lymph node dissection

^a^: Others: palliative treatment for recurrence, complementary treatment or simple follow-up etc

### Factor analysis of needs

As a result of factor analysis for the needs of the breast cancer survivors, all seven factors were extracted and they were referred to as ‘Healthcare staff’, ‘Information and education’, ‘Psychological problem’, ‘Physical symptom’, ‘Social support’, ‘Hospital service’, ‘Religious support’ according the contents of the extracted items. As factor analysis showed that one item (Q51) out of 59 items could not be classified as a specific factor, it was excluded from need variables of breast cancer survivors (Additional file 1: Table S2). Among top 10 unmet needs, the ‘Information and education’ domain included 8 items, and the ‘Healthcare staff’ domain included 2 items (Table 2). The level of unmet need was highest in the domain ‘Information and education’ (mean ± SD; 1.70 ± 1.14) and the item with the highest level of unmet need was ‘Needed help in coping with fear of recurrence’ (2.04 ± 1.09).

**Table 2:** Top 10 unmet needs of study subjects

Rank	Item	Score (Mean ± SD)	Domain
1	Needed help in coping with fear of recurrence	2.04 ± 1.09	Information and education
2	Needed information about correct diet (food to eat, food to avoid)	1.98 ± 1.11	Information and education
3	Needed information about current status of my illness and its future courses	1.84 ± 1.14	Information and education
4	Needed information or education about things that I can do at home for my health	1.82 ± 1.13	Information and education
5	Needed information about tests and treatments	1.80 ± 1.16	Information and education
6	Needed information about symptoms require a hospital visit	1.79 ± 1.12	Information and education
7	Needed help with worries about treatment sequelae	1.79 ± 1.13	Information and education
8	Wished to be able to seek doctor in a quick and easy way when in need	1.78 ± 1.20	Healthcare staff
9	Needed information about financial support for medical expenses from government	1.77 ± 1.18	Information and education
10	Wished my doctor to be easy, specific, and honest in his/her explanation	1.74 ± 1.22	Healthcare staff

### Needs by clinicopathological characteristics

Among age groups, the 50–59 age group showed higher levels of unmet needs in healthcare staff, psychological problem and hospital service and the group with family history of breast cancer had a higher level of hospital service needs. Stage III and IV groups showed higher levels of unmet needs in all domains except healthcare staff, information and education and religious support, the multiplicity group had higher levels of unmet needs in healthcare staff, physical symptom and religious support and HER2 positive breast cancer survivors were found to experience higher levels of unmet needs in social support and hospital service. Patients who underwent target therapy showed higher levels of needs for hospital service and the group of patients receiving palliative treatment for recurrence or complementary treatment showed higher levels of unmet needs in psychological and physical symptoms and social support. However, there were no statistically significant differences in the level of unmet needs according to menopausal status, time since diagnosis, operation method, the presence of estrogen or progesterone receptor, the use of chemotherapy, radiation therapy and hormonal therapy and presence of co-morbidity (Table 3).

**Table 3:** Needs by clinicopathological characteristics of study subjects

Variable	Healthcare staff	Information and education	Psychological problem	Physical symptom	Social support	Hospital service	Religious support	Total
Age (years)								
< 40	1.29 ± 1.04a	1.48 ± 1.00	1.21 ± 1.11a	0.91 ± 0.93ab	0.72 ± 0.74	1.32 ± 1.17a	0.65 ± 1.00	1.06 ± 0.84
40 ~ 49	1.53 ± 0.99ab	1.74 ± 0.95	1.39 ± 0.94a	1.13 ± 0.86bc	0.82 ± 0.72	1.62 ± 0.99b	0.96 ± 1.13	1.26 ± 0.76
50 ~ 59	1.69 ± 0.97b	1.78 ± 0.84	1.47 ± 0.96b	1.28 ± 0.85c	0.96 ± 0.79	1.66 ± 1.04b	0.98 ± 1.16	1.37 ± 0.72
≥ 60	1.28 ± 0.94a	1.53 ± 0.85	1.06 ± 0.81a	0.80 ± 0.69a	0.70 ± 0.69	1.17 ± 0.97a	0.83 ± 1.13	1.04 ± 0.61
*p*-value	0.042	0.170	0.096	0.008	0.155	0.030	0.361	0.030
Menopausal status								
Premenopause	1.53 ± 1.00	1.72 ± 0.93	1.35 ± 0.97	1.09 ± 0.86	0.80 ± 0.70	1.57 ± 1.03	0.90 ± 1.13	1.24 ± 0.75
Postmenopause	1.54 ± 0.98	1.67 ± 0.87	1.37 ± 0.96	1.16 ± 0.87	0.93 ± 0.82	1.50 ± 1.05	0.94 ± 1.12	1.27 ± 0.75
p-value	0.930	0.648	0.866	0.519	0.144	0.519	0.794	0.652
Time since diagnosis (years)								
≤ 1	1.56 ± 1.02	1.85 ± 0.85	1.56 ± 0.97	1.28 ± 0.96	0.93 ± 0.83	1.63 ± 1.07	0.99 ± 1.19	1.35 ± 0.79
> 1, ≤ 3	1.52 ± 0.99	1.71 ± 0.90	1.35 ± 0.96	1.09 ± 0.85	0.86 ± 0.72	1.63 ± 1.00	0.97 ± 1.11	1.27 ± 0.75
> 3, ≤ 5	1.40 ± 0.95	1.54 ± 0.95	1.18 ± 0.94	0.99 ± 0.79	0.70 ± 0.71	1.39 ± 1.05	0.73 ± 1.07	1.09 ± 0.68
> 5	1.59 ± 0.97	1.53 ± 0.94	1.26 ± 0.97	1.13 ± 0.82	0.79 ± 0.74	1.22 ± 1.03	0.77 ± 1.07	1.22 ± 0.76
p-value	0.782	0.172	0.143	0.252	0.324	0.121	0.418	0.235
Family history of breast cancer								
No	1.55 ± 0.98	1.72 ± 0.90	1.37 ± 0.97	1.13 ± 0.86	0.85 ± 0.75	1.59 ± 1.04	0.94 ± 1.13	1.27 ± 0.74
Yes	1.27 ± 1.05	1.45 ± 0.94	1.24 ± 0.96	1.00 ± 0.93	0.77 ± 0.80	1.01 ± 0.97	0.58 ± 0.99	1.08 ± 0.83
*p*-value	0.151	0.138	0.486	0.451	0.567	0.006	0.111	0.209
Operation method of breast								
BCS	1.49 ± 0.99	1.67 ± 0.89	1.30 ± 0.96	1.06 ± 0.86	0.81 ± 0.73	1.50 ± 1.03	0.84 ± 1.10	1.24 ± 0.76
Mastectomy	1.58 ± 0.94	1.77 ± 0.90	1.49 ± 0.96	1.21 ± 0.86	0.92 ± 0.78	1.69 ± 1.03	1.14 ± 1.19	1.41 ± 0.73
(NA)SSM	1.46 ± 1.05	1.55 ± 1.09	1.38 ± 1.00	1.25 ± 0.84	0.81 ± 0.71	1.43 ± 1.11	0.74 ± 1.01	1.23 ± 0.81
*p*-value	0.752	0.579	0.344	0.296	0.556	0.325	0.111	0.253
Operation method of axilla								
SLNB	1.48 ± 1.03	1.65 ± 0.93	1.32 ± 0.98	1.08 ± 0.85	0.84 ± 0.78	1.49 ± 1.04	0.94 ± 1.14	1.26 ± 0.78
ALND	1.57 ± 0.91	1.75 ± 0.85	1.41 ± 0.94	1.19 ± 0.88	0.84 ± 0.68	1.64 ± 1.02	0.85 ± 1.08	1.33 ± 0.72
*p*-value	0.430	0.308	0.444	0.265	0.954	0.218	0.486	0.453
TNM stage								
0	1.31 ± 0.90	1.36 ± 0.82	1.01 ± 0.97a	0.96 ± 0.91	0.49 ± 0.53a	1.08 ± 0.85a	0.42 ± 0.67	0.93 ± 0.61
I	1.44 ± 1.02	1.60 ± 0.97	1.21 ± 0.94ab	0.95 ± 0.78	0.69 ± 0.67ab	1.40 ± 1.01ab	0.90 ± 1.13	1.14 ± 0.74
II	1.60 ± 0.99	1.77 ± 0.83	1.44 ± 0.96ab	1.22 ± 0.88	0.96 ± 0.77b	1.64 ± 1.06b	0.95 ± 1.14	1.32 ± 0.74
III & IV	1.63 ± 0.94	1.82 ± 0.96	1.62 ± 0.97b	1.31 ± 0.96	1.00 ± 0.85b	1.79 ± 1.04b	1.00 ± 1.15	1.42 ± 0.79
*p*-value	0.438	0.187	0.030	0.025	0.004	0.036	0.435	0.034
Multiplicity								
No	1.46 ± 0.98	1.66 ± 0.89	1.31 ± 0.95	1.07 ± 0.84	0.81 ± 0.73	1.50 ± 1.04	0.84 ± 1.08	1.20 ± 0.73
Yes	1.82 ± 0.97	1.86 ± 0.97	1.57 ± 1.01	1.33 ± 0.93	0.99 ± 0.82	1.74 ± 1.03	1.25 ± 1.26	1.48 ± 0.80
*p*-value	0.011	0.117	0.054	0.035	0.088	0.099	0.011	0.007
Estrogen receptor								
Negative	1.44 ± 0.96	1.63 ± 0.93	1.38 ± 0.99	1.16 ± 0.87	0.92 ± 0.80	1.47 ± 1.10	0.96 ± 1.16	1.25 ± 0.76
Positive	1.56 ± 1.00	1.72 ± 0.90	1.35 ± 0.96	1.11 ± 0.86	0.83 ± 0.74	1.57 ± 1.02	0.92 ± 1.12	1.25 ± 0.75
*p*-value	0.360	0.476	0.854	0.697	0.371	0.477	0.804	0.978
Progesterone receptor								
Negative	1.50 ± 0.94	1.71 ± 0.88	1.44 ± 0.95	1.22 ± 0.84	0.92 ± 0.78	1.52 ± 1.04	1.03 ± 1.18	1.31 ± 0.75
Positive	1.55 ± 1.01	1.70 ± 0.92	1.33 ± 0.97	1.08 ± 0.87	0.83 ± 0.74	1.56 ± 1.04	0.88 ± 1.11	1.23 ± 0.75
*p*-value	0.696	0.959	0.346	0.199	0.291	0.773	0.277	0.426
HER2								
Negative	1.48 ± 0.99	1.64 ± 0.93	1.29 ± 0.96	1.07 ± 0.86	0.77 ± 0.70	1.46 ± 1.03	0.86 ± 1.14	1.19 ± 0.74
Positive	1.63 ± 0.98	1.84 ± 0.83	1.52 ± 0.95	1.22 ± 0.86	1.01 ± 0.84	1.73 ± 1.05	1.03 ± 0.10	1.38 ± 0.74
*p*-value	0.214	0.059	0.505	0.139	0.015	0.035	0.220	0.039
Chemotherapy								
No	1.49 ± 1.03	1.53 ± 0.91	1.26 ± 1.00	0.97 ± 0.81	0.75 ± 0.74	1.43 ± 1.02	1.05 ± 1.15	1.21 ± 0.76
Yes	1.52 ± 0.97	1.74 ± 0.90	1.39 ± 0.95	1.16 ± 0.88	0.87 ± 0.74	1.58 ± 1.04	0.86 ± 1.10	1.31 ± 0.76
*p*-value	0.821	0.071	0.298	0.099	0.238	0.283	0.183	0.349
Hormonal therapy								
No	1.53 ± 0.98	1.70 ± 0.92	1.40 ± 0.99	1.21 ± 0.92	0.90 ± 0.80	1.52 ± 1.12	0.85 ± 1.09	1.31 ± 0.78
Yes	1.50 ± 0.99	1.69 ± 0.90	1.34 ± 0.95	1.07 ± 0.84	0.81 ± 0.72	1.55 ± 1.00	0.93 ± 1.13	1.28 ± 0.75
*p*-value	0.801	0.938	0.624	0.201	0.320	0.858	0.585	0.753
Radiation therapy								
No	1.45 ± 0.93	1.69 ± 0.89	1.50 ± 0.92	1.16 ± 0.86	0.92 ± 0.78	1.52 ± 1.08	1.02 ± 1.15	1.32 ± 0.75
Yes	1.52 ± 1.00	1.69 ± 0.91	1.31 ± 0.97	1.10 ± 0.86	0.81 ± 0.73	1.54 ± 1.02	0.87 ± 1.11	1.27 ± 0.76
*p*-value	0.601	0.999	0.168	0.582	0.322	0.880	0.361	0.630
Target therapy								
No	1.48 ± 0.99	1.66 ± 0.92	1.30 ± 0.97	1.06 ± 0.86	0.78 ± 0.72	1.48 ± 1.03	0.87 ± 1.12	1.24 ± 0.75
Yes	1.59 ± 0.99	1.81 ± 0.83	1.53 ± 0.91	1.29 ± 0.86	1.03 ± 0.81	1.77 ± 1.04	1.03 ± 1.12	1.44 ± 0.77
*p*-value	0.462	0.189	0.085	0.056	0.016	0.050	0.312	0.063
Co-morbidity								
No	1.46 ± 0.94	1.78 ± 0.83	1.40 ± 0.94	1.19 ± 0.85	0.86 ± 0.73	1.52 ± 1.00	0.99 ± 1.17	1.28 ± 0.73
Yes	1.52 ± 1.00	1.66 ± 0.93	1.34 ± 0.97	1.07 ± 0.86	0.82 ± 0.73	1.54 ± 1.04	0.91 ± 1.12	1.22 ± 0.75
*p*-value	0.679	0.327	0.635	0.332	0.651	0.908	0.626	0.563
Adjuvant treatment								
During	1.50 ± 1.01	1.81 ± 0.88	1.43 ± 0.98ab	1.21 ± 0.91ab	0.94 ± 0.77b	1.60 ± 1.08	0.99 ± 1.16	1.32 ± 0.77a
After	1.47 ± 0.96	1.58 ± 0.91	1.20 ± 0.91a	0.98 ± 0.76a	0.69 ± 0.67a	1.46 ± 1.00	0.81 ± 1.09	1.13 ± 0.68ab
Others^a^	1.77 ± 0.97	1.75 ± 0.97	1.67 ± 1.01b	1.27 ± 0.98b	1.01 ± 0.81b	1.62 ± 1.04	1.06 ± 1.13	1.43 ± 0.83b
*p*-value	0.165	0.103	0.009	0.039	0.005	0.471	0.260	0.022

Abbreviations: *BCS* breast conserving surgery, (*NA*) (nipple areolar), *SSM* skin sparing mastectomy, *SLNB* sentinel lymph node biopsy, *ALND* axillary lymph node dissection

^a^: Others: palliative treatment for recurrence, complementary treatment or simple follow-up etc

### Needs by sociodemographic characteristics

Regarding the level of needs according to the marital status, the ‘separated’ (divorce, separation or bereavement) group was found to have higher levels of unmet needs in the domains of healthcare staff, information and education, psychological problem and the group without religion had higher levels of unmet needs in the domains of hospital service, psychological problem, hospital service and religious support. The unemployed group experienced higher levels of unmet needs in all domains except healthcare staff and religious support. In addition, there was no significant difference in the level of unmet needs according to the education level, income before and after diagnosis of breast cancer and joining the self-help group (Table 4).

**Table 4:** Needs by sociodemographic characteristics of study subjects

Variable	*n*(%)	Healthcare staff	Information and education	Psychological problem	Physical symptom	Social support	Hospital service	Religious support	Total
Education									
High school	202 (63.3)	1.51 ± 0.94	1.71 ± 0.87	1.39 ± 0.93	1.16 ± 0.86	0.88 ± 0.73	1.53 ± 1.00	0.85 ± 1.09	1.26 ± 0.72
College	117 (36.7)	1.51 ± 1.06	1.64 ± 0.97	1.03 ± 0.87	1.03 ± 0.87	0.77 ± 0.76	1.54 ± 1.09	0.98 ± 1.17	1.20 ± 0.78
*p*-value		0.943	0.501	0.328	0.174	0.219	0.928	0.321	0.498
Marital status									
Single	16(5.0)	1.03 ± 1.12a	1.19 ± 1.03a	0.69 ± 0.94a	0.93 ± 1.02	0.58 ± 0.87a	1.00 ± 1.05a	0.63 ± 1.02	0.84 ± 0.86a
Married	258 (81.4)	1.49 ± 0.97ab	1.67 ± 0.88b	1.33 ± 0.94b	1.10 ± 0.84	0.82 ± 0.72ab	1.52 ± 1.02b	0.91 ± 1.12	1.22 ± 0.72ab
Separated^a^	43 (13.6)	1.75 ± 1.01b	1.95 ± 0.94b	1.66 ± 0.97b	1.20 ± 0.91	1.00 ± 0.71b	1.77 ± 1.03b	1.03 ± 1.19	1.45 ± 0.78b
*p*-value		0.038	0.013	0.002	0.534	0.124	0.038	0.483	0.017
Religion									
Yes	212 (66.9)	1.47 ± 0.97	1.67 ± 0.92	1.25 ± 0.93	1.08 ± 0.85	0.81 ± 0.73	1.44 ± 1.04	1.18 ± 1.21	1.25 ± 0.75
No	105 (33.1)	1.57 ± 1.01	1.71 ± 0.90	1.55 ± 0.98	1.13 ± 0.88	0.88 ± 0.76	1.70 ± 1.00	0.40 ± 0.70	1.21 ± 0.73
*p*-value		0.391	0.711	0.008	0.675	0.428	0.038	≤0.001	0.640
Employment									
Employed	101 (32.4)	1.38 ± 1.03	1.45 ± 0.93	1.11 ± 0.98	0.93 ± 0.84	0.72 ± 0.71	1.38 ± 1.10	0.90 ± 1.12	1.08 ± 0.73
Unemployed	211 (67.6)	1.59 ± 0.96	1.80 ± 0.87	1.67 ± 0.94	1.22 ± 0.87	0.91 ± 0.76	1.65 ± 0.99	0.92 ± 1.12	1.32 ± 0.74
*p*-value		0.072	0.001	0.002	0.007	0.035	0.030	0.866	0.007
Income, in thousand Korean won (before diagnosis)									
< 2000	66 (21.7)	1.46 ± 1.01	1.70 ± 0.91	1.46 ± 0.92	1.17 ± 0.92	0.93 ± 0.78	1.40 ± 1.09	0.75 ± 1.17	1.25 ± 0.77
2000–4000	100 (32.9)	1.48 ± 0.95	1.64 ± 0.88	1.35 ± 0.97	1.12 ± 0.84	0.84 ± 0.71	1.58 ± 0.94	1.00 ± 1.15	1.26 ± 0.73
4000–6000	91 (29.9)	1.50 ± 0.97	1.73 ± 0.94	1.30 ± 0.96	1.03 ± 0.83	0.77 ± 0.75	1.50 ± 1.06	0.86 ± 1.04	1.20 ± 0.76
≥ 6000	47 (15.5)	1.60 ± 1.08	1.63 ± 0.92	1.27 ± 1.05	1.12 ± 0.95	0.77 ± 0.74	1.67 ± 1.14	1.02 ± 1.17	1.24 ± 0.76
*p*-value		0.889	0.903	0.705	0.786	0.563	0.527	0.470	0.956
Income, in thousand Korean won (after diagnosis)									
< 2000	97 (32.2)	1.55 ± 0.97	1.73 ± 0.89	1.53 ± 0.90	1.26 ± 0.91	0.91 ± 0.73a	1.48 ± 1.05	0.88 ± 1.14	1.32 ± 0.75
2000–4000	101 (33.6)	1.48 ± 0.99	1.68 ± 0.86	1.35 ± 0.99	1.09 ± 0.85	0.94 ± 0.79a	1.64 ± 0.97	0.99 ± 1.13	1.26 ± 0.76
4000–6000	70 (23.3)	1.49 ± 0.96	1.64 ± 0.97	1.18 ± 0.93	0.99 ± 0.84	0.62 ± 0.66b	1.44 ± 1.12	0.69 ± 1.00	1.10 ± 0.69
≥ 6000	33 (11.0)	1.45 ± 1.10	1.59 ± 0.94	1.12 ± 1.08	0.95 ± 0.86	0.64 ± 0.68b	1.55 ± 1.10	1.00 ± 1.20	1.12 ± 0.76
*p*-value		0.942	0.846	0.060	0.164	0.012	0.606	0.325	0.220
Self-help group									
Yes	31(9.9)	1.65 ± 0.99	1.69 ± 0.91	1.21 ± 0.89	1.33 ± 0.94	0.96 ± 0.82	1.67 ± 1.09	1.32 ± 1.28	1.36 ± 0.81
No	281 (90.1)	1.49 ± 0.99	1.69 ± 0.90	1.37 ± 0.97	1.09 ± 0.85	0.83 ± 0.74	1.53 ± 1.03	0.86 ± 1.09	1.23 ± 0.74
*p*-value		0.397	0.983	0.371	0.138	0.341	0.500	0.060	0.340

^a^: Separated: divorce, separation, bereavement

### Needs by psychosocial status and QoL

**Fig. 1:**
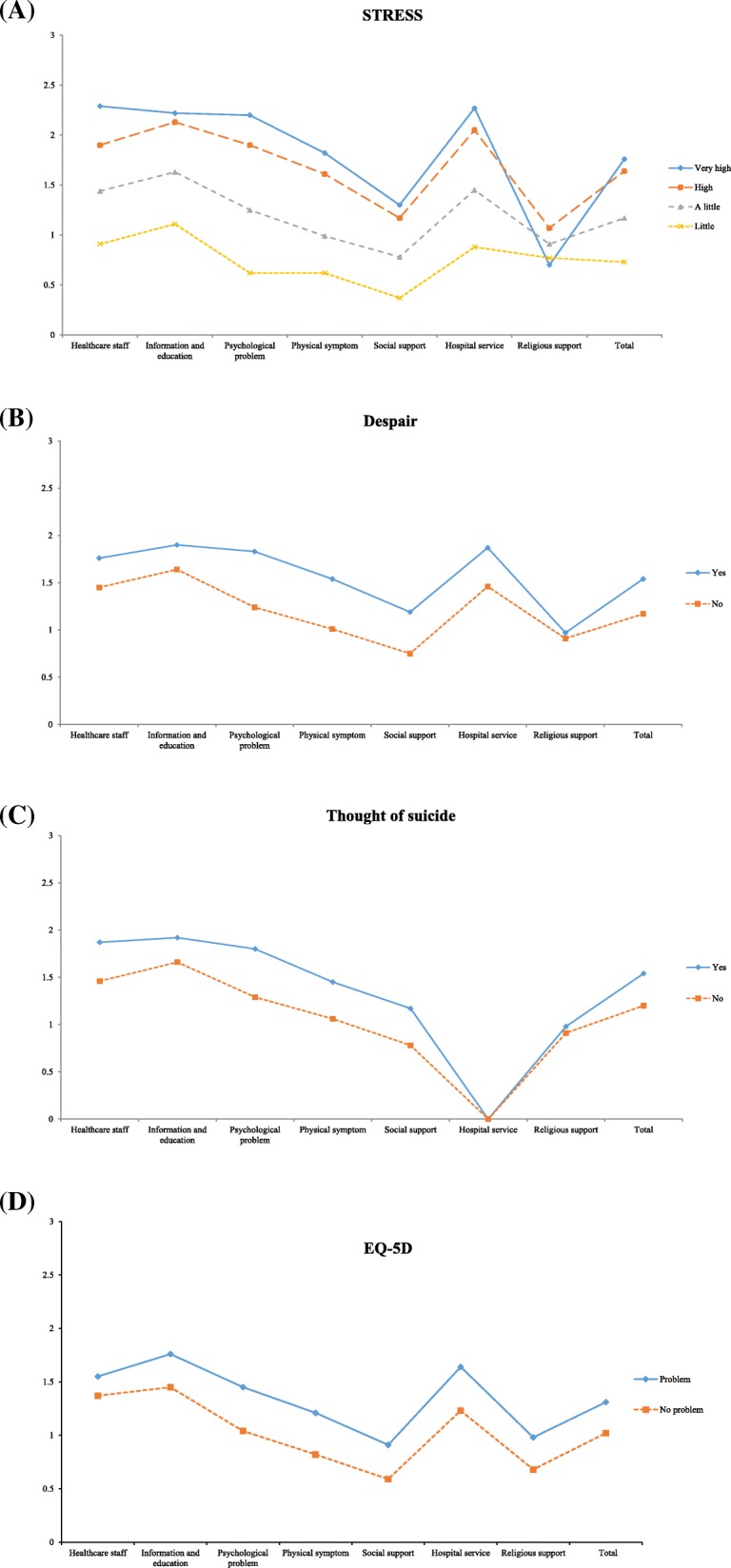
Needs by psychosocial status and quality of life of study subjects. **a** Stress (**b**) Despair (**c**) Thought of suicide (**d**) EQ-5D

Patients with a higher level of stress showed higher levels of unmet needs in all domains except religious support and the group with the experience of despair for more than 2 weeks showed high levels of unmet needs in psychological problem, physical symptom and social support. In addition, the group with thoughts of suicide had higher levels of unmet needs for psychological problems (Fig. 1, Additional file 1: Table S3). The survivors with QoL problems as measured by EQ-5D showed statistically significantly higher levels of unmet needs in all domains except healthcare staff (Fig. 1, Additional file 1: Table S4).

### Multiple regression analysis by needs

Multiple regression analysis was conducted to determine the influence of each independent variable on the total score for each unmet need which was the dependent variable and the results are shown in Table 5.

**Table 5:** The result of multiple regression analysis by needs

Variable	Healthcare staff		Information and education		Psychological problem		Physical symptom		Social support		Hospital service		Religious support	
	β	T	β	T	β	T	β	T	β	T	β	T	β	T
Marital status (ref: Single)														
Married	0.151	0.579	0.217	0.920	0.306	1.294	−0.227	−1.055	0.012	0.061	0.246	0.918	0.139	0.450
Separated etc	0.180	0.592	0.248	0.898	0.279	1.010	−0.403	−1.602	− 0.060	− 0.269	0.384	1.221	0.231	0.626
Religion (ref: Yes)														
No	−0.019	−0.153	− 0.045	− 0.392	0.198	1.727	− 0.036	− 0.342	− 0.038	−0.415	0.163	1.249	−0.737	−4.904
Employment (ref: Yes)														
No	0.216	1.583	0.272	2.202^*^	0.201	1.628	0.171	1.512	−0.012	−0.119	0.174	1.241	−0.233	−1.436
Income (ref: ≥600)														
400–600	0.080	0.374	0.064	0.330	0.102	0.531	0.099	0.563	0.014	0.091	−0.049	−0.222	−0.117	− 0.467
200–400	− 0.039	−0.189	− 0.071	− 0.384	0.127	0.683	0.051	0.300	0.214	1.438	− 0.100	−0.474	0.078	0.320
< 200	−0.118	−0.554	− 0.157	− 0.810	0.164	0.845	0.046	0.259	0.109	0.700	−0.369	−1.678	− 0.189	− 0.746
Age (ref: ≤39)														
40–49	0.299	1.634	0.253	1.523	0.189	1.139	0.284	1.877	0.100	0.747	0.331	1.742	0.245	1.120
50–59	0.339	1.844	0.267	1.597	0.161	0.962	0.381	2.506^*^	0.138	1.024	0.249	1.304	0.291	1.321
≥ 60	0.195	0.786	0.174	0.771	−0.068	− 0.301	0.057	0.276	−0.027	− 0.150	0.094	0.364	−0.006	− 0.021
Family history (ref: No)														
Yes	− 0.196	− 0.928	−0.217	−1.131	− 0.006	−0.029	− 0.053	−0.304	0.012	0.079	−0.412	−1.847	−0.192	− 0.747
TNM stage (ref: 0)														
I	0.325	0.941	0.162	0.515	0.270	0.859	−0.251	−0.876	0.108	0.432	0.462	1.300	0.493	1.207
II	0.440	1.239	0.267	0.829	0.453	1.404	−0.061	−0.208	0.306	1.185	0.611	1.674	0.671	1.594
III + IV	0.280	0.678	0.242	0.644	0.671	1.788	−0.061	− 0.177	0.375	1.251	0.570	1.342	0.592	1.210
Multiplicity (ref: No)														
Yes	0.351	2.265^*^	0.179	1.273	0.187	1.327	0.269	2.092^*^	0.110	0.978	0.190	1.190	0.338	1.838
HER2 (ref: No)														
Yes	0.078	0.374	0.335	1.763	0.283	1.488	0.061	0.349	0.052	0.345	0.242	1.124	−0.017	− 0.069
Breast surgery (ref: BCS)														
Mastectomy	0.106	0.598	0.013	0.080	−0.070	−0.434	− 0.063	−0.429	− 0.075	−0.578	0.127	0.691	0.195	0.922
(NA)SSM	0.057	0.224	−0.188	− 0.810	− 0.140	− 0.601	0.272	1.284	− 0.039	− 0.208	–	–	− 0.157	− 0.520
Axillary surgery (ref: SLNB)														
ALND	0.061	0.419	0.023	0.172	− 0.071	− 0.537	0.017	0.138	− 0.135	−1.281	0.078	0.521	−0.125	− 0.724
Chemotherapy (ref: No)														
Yes	− 0.159	− 1.003	0.153	1.066	− 0.075	− 0.520	0.023	0.176	0.008	0.066	−0.088	− 0.539	− 0.205	− 1.087
Hormonal Tx (ref: No)														
Yes	− 0.047	−0.359	− 0.019	−0.163	− 0.027	−0.226	− 0.177	−1.635	−0.062	− 0.655	0.077	0.569	0.117	0.753
Radiation Tx (ref: No)														
Yes	0.348	1.773	0.181	1.014	−0.084	− 0.471	0.169	1.042	−0.091	− 0.637	0.217	1.071	−0.020	− 0.084
Target Tx (ref: No)														
Yes	− 0.002	− 0.007	− 0.292	−1.348	−0.116	− 0.533	0.080	0.406	0.134	0.772	0.001	0.002	0.205	0.722
Adjuvant treatment (ref: During)														
After	0.142	1.045	−0.042	−0.339	0.082	0.665	−0.010	−0.089	− 0.088	−0.893	0.086	0.620	−0.085	−0.523
others	0.203	1.196	−0.136	−0.884	0.196	1.269	0.029	0.204	0.034	0.274	−0.077	−0.441	0.008	0.042
Stress (ref: Little)														
A little	0.548	2.948^†^	0.544	3.225^†^	0.541	3.202^†^	0.422	2.740^†^	0.402	2.977^†^	0.510	2.668^†^	0.147	0.667
High	1.215	5.271^‡^	1.208	5.778^‡^	1.130	5.404^‡^	1.176	6.166^‡^	0.794	4.743^‡^	1.299	5.478^‡^	0.330	1.212
Very high	1.481	5.074^‡^	1.209	4.565^‡^	1.338	5.048^‡^	1.171	4.847^‡^	0.809	3.821^‡^	1.350	4.502^‡^	0.092	0.265
Despair (ref: No)														
Yes	0.049	0.306	− 0.011	− 0.073	0.276	1.900	0.271	2.046	0.224	1.907	0.165	0.995	0.035	0.180
Thought of suicide (ref: No)														
Yes	0.076	0.423	0.054	0.333	0.092	0.562	−0.029	−0.195^*^	0.105	0.798	−0.065	−0.350	0.028	0.127
EQ5-D (ref: No problem)														
Problem	−0.053	−0.356	0.033	0.242	0.057	0.418	0.090	0.730	0.010	0.096	0.116	0.755	0.185	1.046
Adjusted	0.138		0.158		0.247		0.245		0.167		0.182		0.092	
F-value	2.433		2.687		3.958		3.909		2.794		2.984		1.896	
*P*-value	≤0.001		≤0.001		≤0.001		≤0.001		≤0.001		≤0.001		0.004	

^*^: *p* < 0.05, ^†^: *p* < 0.01, ^‡^: *p* < 0.001

Abbreviations: *BCS* breast conserving surgery, (*NA*) (nipple areolar), *SSM* skin sparing mastectomy, *SLNB* sentinel lymph node biopsy, *ALND* axillary lymph node dissection

The 50s age group showed a higher level of recognition for physical symptom needs and the unemployed group expressed greater needs for information and education. Survivors with multiplicity showed higher levels of needs in the domains of healthcare staff and physical symptom. The stress group showed a higher level of recognition for all needs excluding religious support need. In addition, the group with thoughts of suicide had higher levels of unmet needs for physical symptoms. On the other hand, the differences in the level of unmet needs according to marital status, religion, income, family history, stage, operation method, treatment modality and completion of adjuvant treatment were not statistically significant.

## Discussion

Accurate assessment of the unmet needs of a growing number of cancer survivors worldwide is an important step in developing appropriate interventions to improve QoL. If physicians are aware of the specific unmet needs of each cancer survivor, they can have communication with patients or clients effectively and efficiently, which enables them to perform improved care and effective treatment-related decision making. Therefore, the identification and management of unmet needs is an essential component of high-quality health care for cancer survivors. In the present study, we analyzed the unmet needs and associated factors of Korean breast cancer survivors through a multicenter cross-sectional interview survey.

In the analysis of unmet needs according to the type of cancer, many studies have reported higher levels of unmet needs among breast cancer survivors than other cancer survivors [12, 13]. This is believed to be due to the fact that the majority of breast cancer survivors are women and people of younger ages, who are generally known to have higher levels of unmet needs than other cancer survivors.

In Korean breast cancer survivors, the level of unmet needs was found to be highest in the domain ‘Information and education’ and in the item ‘Needed help in coping with fear of recurrence.’ In Western countries, breast cancer survivors are reported to have high levels of unmet needs for ‘psychological problems’, but in Eastern countries, the level of unmet needs for ‘information and education’ is found to be high [14–17], and the results of this study also support such findings of previous studies. This difference between Western and Eastern countries may be thought of as a product of racial and cultural aspects, but in the past, even in Western countries, the level of unmet needs was highest in the domain ‘Information and education’ [18], so it is thought that the unmet needs for ‘information and education’ have decreased a result of the improvement in information provision and education for cancer survivors. Therefore, more efforts need to be made to fulfill the needs for information and education in Eastern countries as well. Especially in Korea, the outpatient consultation time for a one-on-one personal consultation and information provision is very short and at most 10 min in most medical institutions, which is thought to be one of the reasons why the level of unmet needs for ‘information and education’ is shown to be high. Regarding information needs, increasing the amount of information provided is not always the best solution and since information needs may be a result of cancer survivors’ efforts to actively overcome their anxiety or disease, physicians should also pay attention to the problems that information needs stem from and other needs behind them. Most cancer survivors, including breast cancer survivors, have a very high fear of cancer recurrence which is known to be closely associated with unmet needs and QoL [15, 19]. The results of this study also showed that the level of unmet needs for the item ‘Needed help in coping with fear of recurrence’ was highest.

Age is a meaningful variable that should be kept in mind when caring for breast cancer survivors. In the study of self-assessed unmet needs, the middle-age group (46–53 years old) expressed statistically significantly higher levels of needs than the older group [20]. This study also revealed that the 50–59 age group had higher levels of unmet needs in the domains of healthcare staff, psychological problem and hospital service compared to other age groups. In addition, the group with the family history of breast cancer had greater needs for hospital service, which may be a result of previous experience of a family member’s receiving breast cancer treatment.

In the analysis according to the stage of cancer, the levels of unmet needs in the domains of psychological and physical symptoms, social support and hospital service were higher in the group with later stage cancers than the group with earlier stage cancers. This difference can be attributed to the fact that since cancer patients diagnosed at a later stage undergo longer and more complicated treatment causing side effects, have greater fear of recurrence and have more difficulty in performing activities of daily life, they have more needs for the resources of society and medical institutions to help them and other studies have reported the same results [14, 21].

Similarly, multiplicity and HER2 positive breast cancer survivors are also thought to have higher levels of unmet needs due to the concerns about high recurrence rates. In particular, survivors receiving target therapy for HER2 positive breast cancers seem to have high hospital service needs because they need to visit a hospital as frequently as once every three weeks for a long period of one year. The group receiving palliative treatment for recurrence or complementary treatment was found to have higher levels of unmet needs in the domains of psychological and physical symptoms and social support and this result is consistent with the findings of the study by Harrison JD et al. [13].

The analysis of needs according to the survival time after breast cancer diagnosis showed no statistically significant differences, but the level of unmet needs was high in the group with the survival time of less than one year. These results seem to show that although many needs may occur due to shock, fear, and anxiety during the initial days after cancer diagnosis, and adjuvant treatment may give rise to various needs, the needs are decreased over time as the patient experiences the process of accepting the changes due to cancer and adapting to life [22]. Therefore, aggressive efforts and measures are needed to meet the unmet needs of survivors during the initial stage after cancer diagnosis.

The spouse can exchange information with the cancer survivor and give comfort to her or him by talking together and exchanging opinions, and help the survivor remember the information provided during the consultation with the physician or treatment. In the case of survivors without the spouse, more unmet needs arise because they have to rely only on medical staff. In this study, we found that the group of people without the spouse had higher levels of unmet needs in the domains of healthcare staff, information and education and psychological problem and hospital service and these results were consistent with previous studies [23].

The group without religion showed higher levels of unmet needs in the domains of ‘psychological problem’ and ‘hospital service ’, but they had lower levels of unmet needs in ‘religious support.’ These results are in agreement with those of other studies [24], and they are thought to indicate that survivors with religion rely on their religion and have interest in spiritual issues to cope with their disease.

For cancer survivors, jobs provide emotional stability through good interpersonal relationships and social life as well as economic benefits of income. In fact, many cancer survivors are known to want to return to work when they have fully recovered and feel ready [25]. In this regard, the results of this study also showed that the unemployed group had higher levels of unmet needs in all need domains except for healthcare staff and religious support, and these results are concordant with the findings of other studies [26].

Distress is the most important psychological issue of breast cancer survivors. In agreement with the findings of previous studies, this study also found that patients with a higher stress level experienced higher levels of unmet needs in most need domains [23, 27, 28]. These results may be attributed to the interaction between two factors; in other words, it is thought that higher levels of unmet needs increased levels of distress. Since the distress level during the initial period after breast cancer diagnosis is a predictor of long-term distress and there is a need to identify survivors more susceptible to distress as soon as possible and implement active interventions [29]. Early diagnosis and active interventions for the mental health status of cancer survivors are imperatively required because psychosocial support for stress and despair prevents distress from proceeding to the thought of suicide.

Estimating QoL is the most common method for ascertaining sequelae in cancer survivors, with studies revealing that the most frequently reported concerns are psychological and social [30, 31]. There is increasing evidence that unmet needs can have a detrimental effect on QoL of cancer survivors [17, 32]. This study also showed that the group with QoL problem had statistically significantly higher unmet needs in all domains except healthcare staff. Therefore, knowledge about cancer survivors’ unmet needs is necessary in order to help survivors attain good QoL.

Lastly, multiple regression analysis was carried out using the total score of unmet need as the dependent variable, and the results revealed that the 50–59 age group, unemployment, multiplicity, stress and thoughts of suicide were associated with higher levels of unmet needs. Therefore, if the quality of cancer management is improved by more actively identifying and meeting unmet needs of these breast cancer survivors, it is expected to increase the satisfaction level among breast cancer survivors and lead to better treatment outcomes.

The present study has several limitations. First, multiple regression analysis showed that although the independent variables had a significant relationship, the explanatory power of the model was not high, and this is thought to be due to the small sample size. Second, since this study is a hospital-based study of cancer survivors and only the patients from six medical centers were included in this study, a cautious approach is needed in generalizing the findings of this study to all Korean breast cancer survivors. Third, as a cross-sectional study, this study did not evaluate the cause-effect relationships, and therefore, a longitudinal population-based surveillance study is needed to investigate changes in unmet needs according to the time after breast cancer surgery. Finally, the survivors were recruited by universal sampling, which led to sampling bias. Despites these limitations, this study clarified the specific unmet needs of Korean breast cancer survivors and related factors, and thereby provided the basis for deriving improved treatment outcomes and grounds for providing comprehensive cancer care.

## Conclusion

Most prevalent unmet needs in Korean breast cancer survivors were found in the ‘information and education’ domain. Especially, the 50–59 age group, unemployment, multiplicity, stress and thought of suicide were associated with higher levels of unmet needs. The efforts to accurately identify breast cancer survivors vulnerable for specific unmet needs and satisfy their unmet needs are expected to improve quality of care for breast cancer survivors and ultimately enable precision treatment that improves the treatment outcomes.

## Additional file

Additional file 1: **Table S1.** Questionnaires of the Comprehensive Needs Assessment Tool. **Table S2.** Result factor analysis. **Table S3.** Needs by psychosocial status of study subjects. **Table S4.** Needs by quality of life of study subjects. (DOCX 37 kb)

## Data Availability

The datasets generated and/or analyzed during the current study are not publicly available due owing to data privacy policy at our facility, but are available from the corresponding author on reasonable request.

## Abbreviations

(NA)SSM: Nipple areolar skin sparing mastectomy,
ALND: Axillary lymph node dissection
BCS: Breast conserving surgery
CNAT: Comprehensive Needs Assessment Tool
QoL: Quality of life
SLNB: Sentinel lymph node biopsy
