# Supplementary material for: Unmet needs and related factors of Korean breast cancer survivors: a multicenter, cross-sectional study
Source: BMC Cancer. 2019 Aug 27;19:839. doi: 10.1186/s12885-019-6064-8 (PMC6712787; doi:10.1186/s12885-019-6064-8)
Supplement: Supplementary file 1 — Table S1. Questionnaires of the Comprehensive Needs Assessment Tool. Table S2. Result factor analysis. Table S3. Needs by psychosocial status of study subjects. Table S4. Needs by quality of life of study subjects. (DOCX 37 kb) [file 12885_2019_6064_MOESM1_ESM.docx]

**Additional file 1**

**Table S1. Questionnaires of the Comprehensive Needs Assessment Tool**

| **Variables** | **Questionnaire items** |
| --- | --- |
| Information and education | Q1. Needed information about current status of my illness and its future courses  Q2. Needed information about tests and treatments  Q3. Needed information about symptoms require a hospital visit  Q4. Needed an Easy and accurate explanation about its benefits, side effects and application of current medication  Q5. Needed information or education about things that I can do at home for my health  Q6. Needed guidelines or information about complementary and alternative medicine  Q7. Needed information about correct diet (food to eat, food to avoid)  Q8. Needed information about cancer treating hospitals or clinics and physicians  Q9. Needed information about financial support for medical expenses from government  Q10. Needed information about hospice service |
| Psychological problem | Q11. Needed help with feelings of unidentifiable anxiety  Q12. Needed help in coping with fear of recurrence  Q13. Needed help with worries about treatment sequelae  Q14. Needed help with my concerns for the family  Q15. Needed help with worries that I would become a burden to others around me  Q16. Needed help with depression  Q17. Needed help with feelings of anger, irritability, or nervousness  Q18. Needed help with loneliness or feelings of isolation  Q19. Needed help with accepting role changes at home, at work and/or in society after cancer diagnosis  Q20. Needed help with acceptance of changes in my appearances due to cancer |
| Health care staff | Q21. Wished to be respected and treated as a person by my doctor  Q22. Wished my doctor to be easy, specific, and honest in his/her explanation  Q23. Wished to be able to seek doctor in a quick and easy way when in need  Q24. Wished my family and I to be actively involved in the decision making process in choosing tests or treatment  that I may receive  Q25. Wished my health care staff to be in harmonious collaboration and communication among themselves  Q26. Wished sincere interest and empathy from my nurse  Q27. Wished my nurse to explain treatment or care that was being given to me  Q28. Wished my Nurse to promptly attend to my discomfort and pain |
| Physical symptom | Q29. Needed help with pain  Q30. Needed help with lack of energy and/or fatigue  Q31. Needed help with trouble sleeping or oversleeping  Q32. Needed help with diarrhea or constipation  Q33. Needed help with nausea and/or vomiting  Q34. Needed help with lack of appetite  Q35. Needed help with loss of hair  Q36. Needed help with shortness of breath  Q37. Needed help with my body feeling a sense of numbness and/or tingling  Q38. Needed help with feeling of fever and/or hot flashes  Q39. Needed help with decline in my ability to concentration or memorize  Q40. Needed help with changes in sexual life |
| Hospital service | Q41. Wished for a short waiting period between the reservation and the doctor appointment  Q42. Wished to be treated in a pleasant environment  Q43. Needed rehabilitation medical services to help with functional recovery after treatment  Q44. Needed a designated hospital staff who would be able to provide counseling for any concerns, and guidance  Q45. Needed professional psychological counseling services  Q46. Needed a visit-home nursing service |
| Family/Personal relations problem | Q47. Needed help and support from people close to me  Q48. Needed help with difficulties that arose in family relationships after cancer diagnosis  Q49. Needed help with difficulties that arose in interpersonal relationships after cancer diagnosis |
| Religious/Spiritual support | Q50. Needed religious support  Q51. Needed help in finding the meaning of my situation and in coming to terms with it |
| Social support | Q52. Needed an opportunity to share experiences or information with other patients  Q53. Needed counseling and support for my return to work or for reemployment  Q54. Needed transportation services for getting to and from the hospital  Q55. Needed treatment near my home  Q56. Needed lodging services near hospital I was treated in  Q57. Needed help with my economic burden due to cancer  Q58. Needed someone to help me with housekeeping and/or child care  Q59. Needed assisted care in the hospital or at home |

**Table S2. Result factor analysis**

| **Items** | **Healthcare staff** | **Information and education** | **Psychological problem** | **Physical symptom** | **Social support** | **Hospital service** | **Religious support** |
| --- | --- | --- | --- | --- | --- | --- | --- |
| Q27 | **.821** | .301 | .158 | .212 | .166 | .072 | .065 |
| Q28 | **.812** | .275 | .157 | .251 | .187 | .079 | .021 |
| Q26 | **.799** | .262 | .208 | .245 | .191 | .097 | .083 |
| Q25 | **.789** | .270 | .238 | .213 | .193 | .113 | .069 |
| Q22 | **.755** | .424 | .156 | .157 | .175 | .057 | -.010 |
| Q23 | **.739** | .389 | .189 | .159 | .159 | .077 | .033 |
| Q21 | **.739** | .348 | .202 | .205 | .217 | .074 | .140 |
| Q24 | **.713** | .260 | .259 | .164 | .153 | .102 | .088 |
| Q42 | **.612** | .316 | .097 | .298 | .221 | .180 | .200 |
| Q29 | **.461** | .379 | .213 | .456 | .244 | .167 | -.004 |
| Q41 | **.456** | .311 | .140 | .139 | .196 | .330 | .081 |
| Q47 | **.431** | .254 | .337 | .210 | .362 | .273 | .191 |
| Q2 | .379 | **.740** | .118 | .197 | .134 | -.033 | .103 |
| Q1 | .357 | **.740** | .158 | .163 | .109 | -.015 | .166 |
| Q3 | .373 | **.718** | .187 | .177 | .111 | -.036 | .084 |
| Q7 | .295 | **.698** | .198 | .169 | .174 | .212 | -.014 |
| Q5 | .315 | **.690** | .199 | .176 | .171 | .203 | .087 |
| Q4 | .336 | **.685** | .165 | .227 | .113 | .057 | .187 |
| Q6 | .120 | **.678** | .207 | .142 | .108 | .315 | .118 |
| Q8 | .398 | **.672** | .191 | .200 | .107 | .018 | .076 |
| Q13 | .245 | **.653** | .401 | .228 | .157 | .204 | -.090 |
| Q12 | .228 | **.625** | .411 | .138 | .191 | .217 | -.152 |
| Q9 | .344 | **.567** | .251 | .174 | .233 | .139 | -.096 |
| Q10 | .141 | **.418** | .252 | .258 | .205 | -.142 | .298 |
| Q17 | .235 | .236 | **.780** | .259 | .163 | .095 | .056 |
| Q16 | .173 | .285 | **.772** | .226 | .149 | .137 | .092 |
| Q18 | .157 | .206 | **.751** | .290 | .265 | .100 | .108 |
| Q19 | .266 | .269 | **.680** | .166 | .299 | .069 | .088 |
| Q15 | .199 | .376 | **.633** | .169 | .295 | .071 | .076 |
| Q20 | .266 | .268 | **.612** | .242 | .323 | .008 | -.044 |
| Q14 | .249 | .473 | **.560** | .221 | .249 | .117 | .049 |
| Q11 | .190 | .518 | **.547** | .191 | .132 | .185 | -.119 |
| Q51* | .229 | .093 | **.397** | .286 | .369 | .323 | .365 |
| Q34 | .128 | .220 | .205 | **.695** | .389 | .052 | -.041 |
| Q36 | .209 | .163 | .100 | **.685** | .343 | .044 | .094 |
| Q33 | .147 | .299 | .128 | **.675** | .375 | -.004 | .005 |
| Q35 | .198 | .225 | .283 | **.631** | .259 | -.042 | .027 |
| Q32 | .251 | .269 | .268 | **.601** | .257 | .081 | -.059 |
| Q40 | .250 | .065 | .205 | **.587** | .106 | .083 | .330 |
| Q38 | .255 | .168 | .292 | **.561** | .126 | .275 | .247 |
| Q37 | .305 | .264 | .173 | **.556** | .262 | .282 | -.062 |
| Q39 | .240 | .201 | .281 | **.513** | .164 | .331 | .071 |
| Q31 | .374 | .278 | .340 | **.511** | .124 | .279 | .038 |
| Q30 | .359 | .304 | .298 | **.500** | .193 | .346 | -.051 |
| Q54 | .127 | .067 | .138 | .253 | **.782** | .101 | -.066 |
| Q56 | .089 | .143 | .042 | .190 | **.679** | .072 | .094 |
| Q55 | .282 | .220 | .131 | .114 | **.640** | -.031 | .131 |
| Q48 | .272 | .038 | .322 | .335 | **.602** | .122 | .123 |
| Q59 | .140 | .110 | .148 | .284 | **.588** | -.068 | .177 |
| Q53 | .108 | .040 | .269 | .063 | **.582** | .227 | .171 |
| Q57 | .096 | .242 | .217 | .212 | **.578** | .052 | -.139 |
| Q49 | .294 | .052 | .400 | .353 | **.536** | .107 | .157 |
| Q52 | .207 | .339 | .191 | .116 | **.531** | .360 | .036 |
| Q58 | .166 | .131 | .339 | .157 | **.477** | .194 | .110 |
| Q46 | .214 | .157 | .103 | .368 | **.442** | .005 | .307 |
| Q45 | .270 | .261 | .411 | .217 | .294 | **.505** | .130 |
| Q43 | .274 | .294 | .203 | .335 | .313 | **.499** | .121 |
| Q44 | .474 | .375 | .231 | .247 | .194 | **.481** | .084 |
| Q50 | .162 | .143 | .064 | .069 | .314 | .118 | **.686** |

* Items excluded from factor analysis.

**Table S3. Needs by psychosocial status of study subjects**

| Variable | n(%) | Healthcare staff | Information and education | Psychological problem | Physical symptom | Social support | Hospital service | Religious support | Total |
| --- | --- | --- | --- | --- | --- | --- | --- | --- | --- |
| **Stress** |  |  |  |  |  |  |  |  |  |
| Very high | 20(6.2) | 2.29±0.82a | 2.22±0.66a | 2.20±0.75a | 1.82±0.81a | 1.30±0.81a | 2.27±0.88a | 0.70±0.98 | 1.76±0.60 |
| High | 60(18.5) | 1.90±0.92a | 2.13±0.75a | 1.90±0.80a | 1.61±0.85a | 1.17±0.81a | 2.05±0.89a | 1.07±1.11 | 1.64±0.70 |
| A little | 205(63.3) | 1.44±0.96c | 1.63±0.89b | 1.25±0.92b | 0.99±0.80b | 0.78±0.68b | 1.45±1.00b | 0.91±1.13 | 1.17±0.71 |
| Little | 39(12.0) | 0.91±0.89c | 1.11±0.92c | 0.62±0.79c | 0.62±0.71c | 0.37±0.54c | 0.88±0.93c | 0.77±1.18 | 0.73±0.62 |
| p-value |  | **≤0.001** | **≤0.001** | **≤0.001** | **≤0.001** | **≤0.001** | **≤0.001** | 0.479 | **≤0.001** |
| **Despair (≥ 2 weeks)** |  |  |  |  |  |  |  |  |  |
| Yes | 63(19.4) | 1.76±0.99 | 1.90±0.88 | 1.83±0.91 | 1.54±0.89 | 1.19±0.79 | 1.87±1.03 | 0.97±1.11 | 1.54±0.77 |
| No | 261(80.6) | 1.45±0.98 | 1.64±0.91 | 1.24±0.94 | 1.01±0.83 | 0.75±0.70 | 1.46±1.02 | 0.91±1.13 | 1.17±0.73 |
| p-value |  | 0.025 | 0.047 | **≤0.001** | **≤0.001** | **≤0.001** | 0.005 | 0.703 | **≤0.001** |
| **Thought of suicide** |  |  |  |  |  |  |  |  |  |
| Yes | 44(13.6) | 1.87±0.86 | 1.92±0.71 | 1.80±0.79 | 1.45±0.81 | 1.17±0.75 | 1.85±1.02 | 0.98±1.14 | 1.54±0.65 |
| No | 279(86.4) | 1.46±0.99 | 1.66±0.93 | 1.29±0.97 | 1.06±0.86 | 0.78±0.72 | 1.50±1.03 | 0.91±1.13 | 1.20±0.75 |
| p-value |  | 0.011 | 0.035 | **≤0.001** | 0.005 | 0.002 | 0.037 | 0.716 | **0.006** |

**Table S4.** **Needs by quality of life of study subjects**

| Variable | n(%) | Healthcare staff | Information and education | Psychological problem | Physical symptom | Social support | Hospital service | Religious support | Total |
| --- | --- | --- | --- | --- | --- | --- | --- | --- | --- |
| **EQ5-D** |  |  |  |  |  |  |  |  |  |
| No problem | 75(23.4) | 1.37±1.06 | 1.45±1.01 | 1.04±0.92 | 0.82±0.80 | 0.59±0.63 | 1.23±1.07 | 0.68±1.05 | 1.02±0.73 |
| Problem | 245(76.6) | 1.55±0.96 | 1.76±0.86 | 1.45±0.96 | 1.21±0.86 | 0.91±0.76 | 1.64±1.00 | 0.98±1.13 | 1.31±0.74 |
| p-value |  | 0.169 | **0.018** | **0.001** | **0.001** | **≤0.001** | **0.003** | **0.046** | **0.003** |
